# Design of Ctenophore Ca^2+^-Regulated Photoprotein Berovin Capable of Being Converted into Active Protein Under Physiological Conditions: Computational and Experimental Approaches

**DOI:** 10.3390/life14111508

**Published:** 2024-11-19

**Authors:** Ludmila P. Burakova, Nikita V. Ivanisenko, Natalia V. Rukosueva, Vladimir A. Ivanisenko, Eugene S. Vysotski

**Affiliations:** 1 Photobiology Laboratory, Institute of Biophysics of Siberian Branch of the Russian Academy of Sciences, Federal Research Center “Krasnoyarsk Science Center” of Siberian Branch of the Russian Academy of Sciences, Krasnoyarsk 660036, Russia; burakoval@mail.ru; 2 Institute of Fundamental Biology and Biotechnology, Siberian Federal University, Krasnoyarsk 660041, Russia; nata.rukosueva.00@mail.ru; 3 Institute of Cytology and Genetics of the Siberian Branch of the Russian Academy of Sciences, Novosibirsk 630090, Russia; n.ivanisenko@gmail.com (N.V.I.); salix@bionet.nsc.ru (V.A.I.); 4 Novosibirsk State University, Novosibirsk 630090, Russia; 5 AIRI, Moscow 123112, Russia

**Keywords:** bioluminescence, coelenterazine, photoprotein, berovin, ctenophore, molecular modeling, AlphaFold

## Abstract

Here, we describe (1) the AlphaFold-based structural modeling approach to identify amino acids of the photoprotein berovin that are crucial for coelenterazine binding, and (2) the production and characterization of berovin mutants with substitutions of the identified residues regarding their effects on the ability to form an active photoprotein under physiological conditions and stability to light irradiation. The combination of mutations K90M, N107S, and W103F is demonstrated to cause a shift of optimal conditions for the conversion of apo-berovin into active photoprotein towards near-neutral pH and low ionic strength, and to reduce the sensitivity of active berovin to light. According to the berovin spatial structure model, these residues are found in close proximity to the 6-(*p*-hydroxy)-phenyl group of the coelenterazine peroxyanion.

## 1. Introduction

A lot of marine luminous organisms such as radiolarians, ctenophores, coelenterates, squids, crustaceans, etc., employ imidazopyrazinone-type compounds as substrates of light emission reactions [1,2]. The reaction chemical mechanism is most likely the same in organisms utilizing this type of substrates, though it may vary in details based on the reaction-catalyzing enzymes. The luminous cnidarians and ctenophores, whose bioluminescence is caused by Ca^2+^-regulated photoproteins stand out among these other organisms. These proteins are monomeric single-chain globular polypeptides containing a preoxygenated coelenterazine molecule which is tightly bound within the protein inner cavity [1,3,4]. The binding of calcium ions to the protein induces the decarboxylation of preoxygenated coelenterazine. This results in the elimination of one mole of CO_2_ and generation of the product, coelenteramide, in an excited state. Its transition to the ground state is accompanied by blue light emission in the 465–495 nm range [3,4].

Many species of cnidarians and ctenophores are found almost everywhere in the oceans, and most of them are luminous [5,6]. Still, the cDNA sequences are available for only four cnidarian photoproteins (aequorin [7,8,9,10,11], mitrocomin [12,13], clytin [14,15,16,17], and obelins [18,19,20] from the corresponding Aequorea, Mitrocoma, Clytia, and Obelia species) and four ctenophore photoproteins (berovin [21,22], bolinopsin [23], mnemiopsin [24,25], and bathocyrovin [26] from Beroe, Bolinopsis, Mnemiopsis, and Bathocyroe species, respectively). The amino acid sequences of all photoproteins reveal the three canonical sequences composed of the 12 contiguous residues typical for the EF-hand Ca^2+^-binding sites [27,28]. The degree of identity of amino acid sequences between ctenophore and cnidarian photoproteins, however, turned out to be only 29.4% [4].

To date, the spatial structures of cnidarian photoproteins have been determined for the four ligand-dependent conformational states: (i) aequorin [29], obelin [30], clytin [31], and mitrocomin [13] bound with the 2-hydroperoxy adduct of coelenterazine; (ii and iii) obelin bound with the reaction product, coelenteramide, with [32] or without [33] Ca^2+^; and (iv) apo-obelin and apo-aequorin bound with Ca^2+^ [34]. In contrast, the three-dimensional structures of ctenophore photoproteins were determined only for the apoproteins bound with different ions. These are apo-berovin with Ca^2+^ [35] or Mg^2+^ [36] and apo-mnemiopsin loaded with Cd^2+^ [37]. Despite the low identity of amino acid sequences between ctenophore and cnidarian photoproteins, their overall spatial structures appeared to be practically identical [4]. For instance, the alignment of spatial structures of apo-berovin and apo-aequorin showed the RMSD of the main chain atoms to be only 1.77 Å [35]. At the same time, considering the low identity of their amino acid sequences, the residues involved in the formation of the inner substrate-binding cavities and, consequently, in the stabilization of the peroxy adduct of coelenterazine, emitter formation, and catalyzing the bioluminescence reaction in ctenophore and cnidarian photoproteins, are completely different. This certainly accounts for some distinctions in the properties of ctenophore and cnidarian photoproteins.

Among these, a loss of bioluminescence ability on the exposure to light over its entire absorption spectrum is the most important feature [1]. The product formed as a result of berovin photoinactivation was isolated and its chemical structure was determined [38]. It was proposed that photoinactivation may be conditioned by the specific manner of preoxygenated coelenterazine binding in the substrate-binding cavity which, according to the berovin model, may be present as a 2-peroxy anion [39]. Of note is that the specific activity of ctenophore photoproteins is almost half of that of cnidarian photoproteins. It may also be associated with a different mode of preoxygenated coelenterazine binding as well as with the amino acid environment within the inner cavity, which does not promote an efficient bioluminescence reaction [40,41].

In addition, the absorption maximum in the visible region of ctenophore photoproteins is at 435–437 nm, whereas that of cnidarian photoproteins ranges from 460 to 470 nm [1,22]. The TD-DFT studies showed that the distinction in the absorption spectra may be attributed to the difference in the dihedral angles of the 6-(*p*-hydroxy)-phenyl substituent relative to the coelenterazine imidazopyrazinone core [42]. Another feature distinguishing the cnidarian and ctenophore photoproteins is the conditions for converting apoproteins into active photoproteins in solution. The ctenophore apophotoproteins are effectively converted into active proteins only at alkaline pH and high ionic strength [1,22]. By contrast, the formation of active cnidarian photoproteins occurs at physiological pH and ionic strength characteristic of eukaryotic cells [1,43]. It is obvious that these distinctions are also caused by the differences in amino acids that form the inner cavities of these photoproteins.

In this study, we report the construction and properties of berovin mutants that can be converted into active proteins at physiological pH and low ionic strength.

## 2. Materials and Methods

### 2.1. Materials

Tersus DNA polymerase was purchased from Evrogen (Moscow, Russia) and used according to the manufacturer’s instructions. Oligonucleotides were purchased from CCU “Genomika” (Novosibirsk, Russia). Coelenterazine (CTZ) was obtained from NanoLight Technology, a division of Prolume Ltd. (Pinetop, AZ, USA). Other chemicals, unless otherwise stated, were from Sigma-Aldrich and of the purest grade available.

### 2.2. Molecular Modeling

The prediction of various conformational states of the berovin structure was performed using the OpenFold implementation of the AlphaFold model, by providing templates of homologous proteins corresponding to the specific conformational states [44,45]. The OpenFold implementation, available at https://github.com/aqlaboratory/openfold (accessed on 1 June 2023), utilized the ‘model-2-ptm’ AlphaFold weights. In this protocol, the template was employed to predict the berovin structure without the use of Multiple Sequence Alignment (MSA). Template features were input only during the first cycle of AlphaFold model inference, while three recycling rounds were performed to obtain the final structural model.

The impact of mutations on the protein structure was evaluated using the pLDDT (predicted LDDT) confidence metrics derived from the OpenFold model inference. This was done by comparing the difference in pLDDT scores at positions 90, 107, and 107 between the wild-type and mutant structures. The structure of berovin was initially predicted using OpenFold with aequorin as a template (PDB ID: 1EJ3). This predicted structure then served as the template for introducing the mutations by the same protocol. Prior to this, the side chains of the amino acid positions within the template were removed.

The apo-berovin structure was predicted using the ColabFold implementation of AlphaFold [46], with default settings.

### 2.3. Molecular Biology

Site-directed mutagenesis was carried out on the pET22b-BA plasmid [22] for *Escherichia coli* expression carrying the *Beroe abyssicola* wild-type apo-berovin gene. The mutations resulting in the desired amino acid changes were performed by PCR using specific primers and Tersus DNA polymerase. The resulting plasmids were verified by DNA sequencing (SB RAS Genomics Core Facility, Novosibirsk, Russia).

### 2.4. Protein Expression and Purification

For apophotoprotein production, the transformed *E. coli* BL21 (DE3) Codon Plus (RIPL) cells were cultivated with vigorous shaking at 37 °C in LB medium containing ampicillin (200 μg mL^−1^). Protein expression was induced with 1 mM IPTG at OD600 of 0.6–0.8 and the cultivation was continued for another 3 h. Apoproteins were purified as previously described for recombinant wild-type berovin [22]. Optimal activation conditions were determined for each mutant with respect to pH and ionic strength. Each mutant was activated by coelenterazine under proper conditions during 24 h and then purified. Coelenterazine concentration in the methanol stock solution was determined spectrophotometrically (ε_435 nm_ = 9800 M^−1^ cm^−1^) [1]. The active photoprotein was separated from apophotoprotein and coelenterazine excess via ion-exchange chromatography by applying a linear gradient of NaCl from 0 M to 0.5 M on a Capto HiRes Q 5.50 column (GE Healthcare, Chicago, IL, USA) with a flow rate of 1 mL/min. The elution buffers were as follows: A—5 mM EDTA, 20 mM Tris-HCl pH 7.2; and B—1 M NaCl, 5 mM EDTA, 20 mM Tris-HCl pH 7.2. Freshly purified proteins were immediately used in the experiments. The active photoprotein yield was estimated using the following equation:

(1) $$Y(\%)=\frac{C_{active}}{C_{active}+C_{apoprotein}}\times 100\%$$

where *C_active_* and *C_apoprotein_* are protein concentrations in the fraction corresponding to the active photoprotein and apophotoprotein obtained by chromatography on a Capto HiRes Q 5.50. The protein concentration was determined with the Dc Bio-Rad protein assay kit (Bio-Rad, Hercules, CA, USA). All procedures with the active wild-type berovin and its mutants including bioluminescence and spectral measurements were performed in the dark or under dim red light to avoid photoinactivation. The yield of the active photoprotein was estimated by averaging of at least two independent protein preparations.

### 2.5. Determination of Apparent Dissociation Constant of the Apophotoprotein–Coelenterazine Complex

The apparent dissociation constant of the apophotoprotein–coelenterazine complex was determined using the quenching of apophotoprotein Trp fluorescence upon binding to coelenterazine as previously described [47]. Fluorescence was measured with a Varian Cary Eclipse spectrofluorometer (Agilent Technologies, Santa Clara, CA, USA) at 20 °C in 5 mM EDTA, 20 mM bis-Tris-propane-HCl at pH and salt concentration optimal for each apoprotein. To assess fluorescence quenching, only the changes in fluorescence intensity at 336 nm were taken into account. All of the spectra were recorded using a standard quartz cuvette (1 × 1 cm) in a 1 mL initial volume with varied coelenterazine additions in 1 to 5 μL portions up to saturation and were corrected with a computer program supplied with the instrument. The fluorescence intensities were corrected for dilution due to the addition of coelenterazine, for methanol’s influence on Trp fluorescence, for scattered light, and for inner filter effects of the protein and coelenterazine added. To evaluate the inner filter effects, the absorbance was measured at excitation and emission wavelengths and fluorescence (*F*) was corrected using the following equation:

(2) $$F=F_{unc}e^{\frac{A_{235}+A_{336}}{2}}$$

where *A*_295_ and A_336_ are the absorbance of the protein and the ligand at excitation and emission wavelengths, respectively, and *F*_unc_ is the uncorrected fluorescence.

The analysis assumes the fraction of the bound ligand to be equal to the ratio of fluorescence quenching (*Q* = *F*_o_ − *F*_q_) to maximum quenching (*Q*_max_ = *F*_o_ − *F*_qmax_), where F_o_, F_q_, and *F*_qmax_ are fluorescence intensities at 336 nm measured in the absence of added ligand, the quenched fluorescence intensity in the presence of ligand, and the maximum fluorescence quenching at a saturating level of ligand, respectively. The apparent dissociation constants were calculated by fitting the relative fluorescence emission to the following equation [48]:

(3) $$\frac{Q}{Q_{\max}}=\frac{(C+L+K_{D})-\sqrt{{\left(C+L+K_{D}\right)}^{2}}-4CL}{2}$$

where *C*, *L*, and *K_D_* are the apophotoprotein and coelenterazine concentrations, and apparent dissociation constant, respectively. The concentrations of apo-berovin and its mutants in these experiments were determined using the corresponding molar extinction coefficients at 280 nm calculated with the Protein Parameters tool (https://protparam.net/index.html, accessed on 1 April 2024) that uses Edelhoch’s method [49].

### 2.6. Bioluminescence Assay

The bioluminescence was measured with a luminometer (BLM-8812, Krasnoyarsk, Russia) by the rapid injection of 10 μL of photoprotein solution in 5 mM EDTA, 20 mM Tris-HCl pH 7.2 into a luminometer cell containing 490 μL of 2 mM CaCl_2_ in 50 mM Tris-HCl pH 8.5 at room temperature. The light signal was recorded until it completely ceased.

Specific bioluminescence activity (L_specific_) was estimated as the ratio of total light determined by integrating bioluminescent signal to protein concentration in a sample by averaging five independent measurements.

The Ca^2+^-independent luminescence (L_Ca-free_) [50] was calculated as a ratio of the maximal light signal from 500 μL of photoprotein in 0.3 M NaCl, 5 mM EDTA, 20 mM Tris-HCl pH 7.2 normalized to protein concentration to the specific bioluminescence activity [20].

### 2.7. Spectral Measurements

Bioluminescence and fluorescence spectra were recorded with a Varian Cary Eclipse spectrofluorometer (Agilent Technologies, Santa Clara, CA, USA). The slit width was 5 nm. To record the bioluminescence spectra, the 100 μL purified protein in ~0.3 M NaCl, 5 mM EDTA, 20 mM Tris-HCl pH 7.2 was placed into the cell of a spectrofluorometer containing 900 μL of 50 mM bis-Tris-propane pH 7.0. Bioluminescence was initiated by the injection of 40 μL of 100 mM CaCl_2_ solution into the same buffer. The light emission spectra were recorded in the range of 370–600 nm with a rate of 12,000 nm/min. The bioluminescence spectra were recorded at an approximately constant light level during the spectral scan. In cases when a substantial change in bioluminescence intensity took place during the spectral measurements, the data points were also corrected for bioluminescence decay.

The fluorescence spectra of Ca^2+^-discharged photoproteins were recorded after the bioluminescence reaction ceased. The measurements were performed in the range of 370–600 nm with a rate of 1200 nm/min. The bioluminescence and fluorescence spectra were corrected for the spectral sensitivity of the detector using an algorithm supplied with the instrument.

### 2.8. Photo- and Thermoinactivation

Photoinactivation was performed for 1 h at 0 °C (on ice) with an incandescent lamp. The 200 μL protein solution with a final concentration of 0.1 mg mL^−1^ in 30 mM NaCl, 5 mM EDTA, 20 mM Tris-HCl pH 7.2 was placed into a 0.5 mL Eppendorf-type tube about 10 cm away from the light source.

Thermal inactivation was performed for 1 h at 37 °C. The 200 μL protein solution with a final concentration of 0.1 mg mL^−1^ in 30 mM NaCl, 5 mM EDTA, 20 mM Tris-HCl pH 7.2 was placed into a 0.5 mL Eppendorf type tube in a dry air thermostat (Binder KB53, Tuttlingen, Germany).

In both cases, bioluminescence was measured every 10 min as described above.

## 3. Results and Discussion

### 3.1. Molecular Modeling

The AlphaFold-predicted structure using ColabFold default settings [46] showed high similarity (RMSD < 1.0 Å) to the experimentally determined berovin structure (PDB ID: 5BPJ) but also included protein fragments not resolved in the X-ray crystal structure. Based on a high similarity of the computed and experimental structures we can reasonably propose that the AlphaFold-produced structure using the default settings corresponds to the one of berovin in apo-state. To identify amino acid residues responsible for the non-physiological conversion of ctenophore apophotoprotein into its active form, we superimposed the spatial model of apo-berovin onto the crystal structure of aequorin in the 2-hydroperoxycoelenterazine-bound state (PDB ID: 1EJ3). The model showed a notably different amino acid composition of the substrate-binding pocket of apo-berovin as compared to aequorin or obelin, suggesting that the residues involved in a proper accommodation of the coelenterazine molecule within the inner cavity, its conversion into peroxy adduct, and its stabilization within the substrate-binding pocket before Ca^2+^-induced bioluminescence differ from those in hydromedusan photoproteins. For instance, the putative substrate-binding site of berovin contains no histidine residues, which, in hydromedusan photoproteins, are involved in the stabilization of 2-hydroperoxycoelenterazine, the decarboxylation of 2-hydroperoxycoelenterazine, and emitter formation [3,51,52,53]. Within the putative berovin substrate-binding pocket, we identified a cluster of polar residues, including Lys90, which can form hydrogen bonds with the side chain of Asn107 and its backbone carbonyl oxygen, all set within a hydrophobic environment. Given that Lys90 is positively charged at neutral pH and is neutral at alkaline pH, we hypothesized that this cluster may account for the alkaline pH for the conversion of apo-berovin into an active photoprotein. Another hypothesis could be that the positively charged Lys90 residue negatively affects the folded state of apo-berovin. In the apo-model, Lys90 forms two hydrogen bonds and lacks negatively charged residues in its vicinity, proposing thereby the structural preference of the Lys90 neutral state.

**Figure 1:**
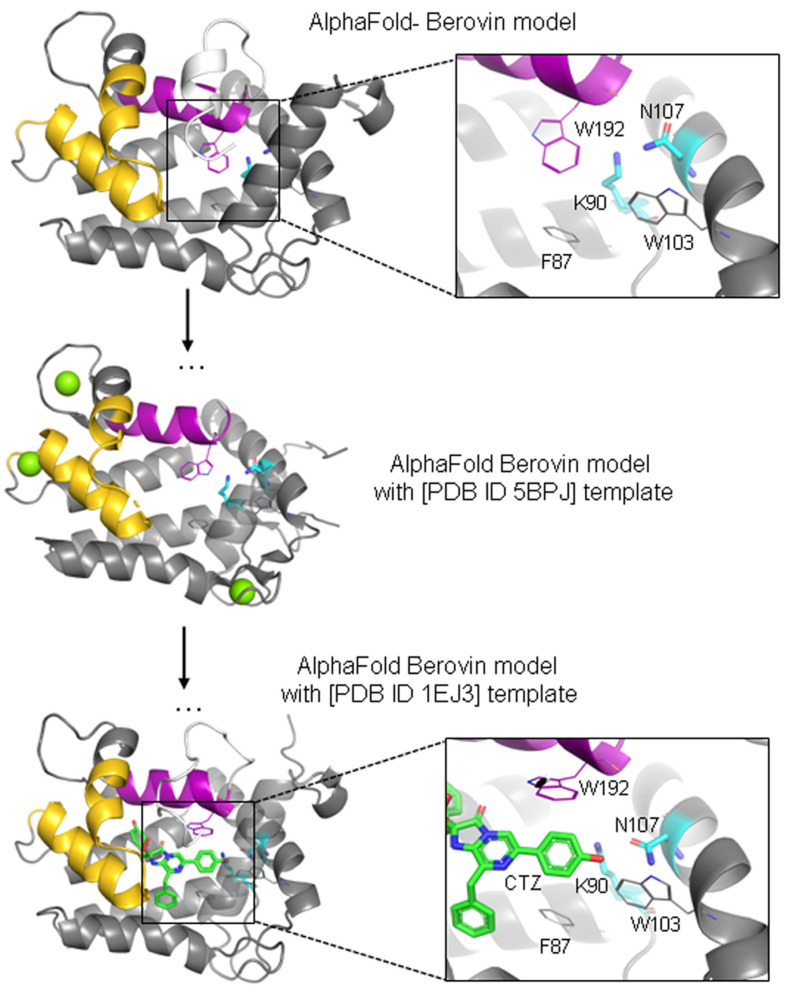
Three-dimensional molecular model of berovin, a ctenophore photoprotein, predicted using AlphaFold with and without MSA. The PDB identifiers for apo-berovin bound with calcium ions (green balls) (PDB ID: 5BPJ) and aequorin bound with 2-hydroperoxycoelenterazine (PDB ID: 1EJ3) used as template structures for the no-MSA calculation are indicated. The mobile regions corresponding to the residues 142–167 and 183–193 are, respectively, highlighted in purple and gold. CTZ, 2-hydroperoxycoelenterazine.

To further elucidate the potential role of the Lys90/Asn107 cluster, we devised a model of berovin based on the structure of aequorin bound with 2-hydroperoxycoelenterazine (PDB ID: 1EJ3) as a template, predicted using the AlphaFold model without MSA (Figure 1). This approach predicts the structural model of berovin in a conformation that is close to the substrate-bound aequorin structure. It highlights several conformational changes the berovin might undergo upon coelenterazine binding, including a rotation of the C-terminal helix (residues 183–193), which may facilitate coelenterazine accommodation. Additionally, assuming that the substrate binding in aequorin is similar to that in berovin (Figure 1), the side chains of the Lys90/Asn107 cluster residues might also form hydrogen bonds with the OH group of the 6-(*p*-hydroxy)-phenyl substituent of coelenterazine, potentially affecting the physicochemical properties of berovin.

**Figure 2:**
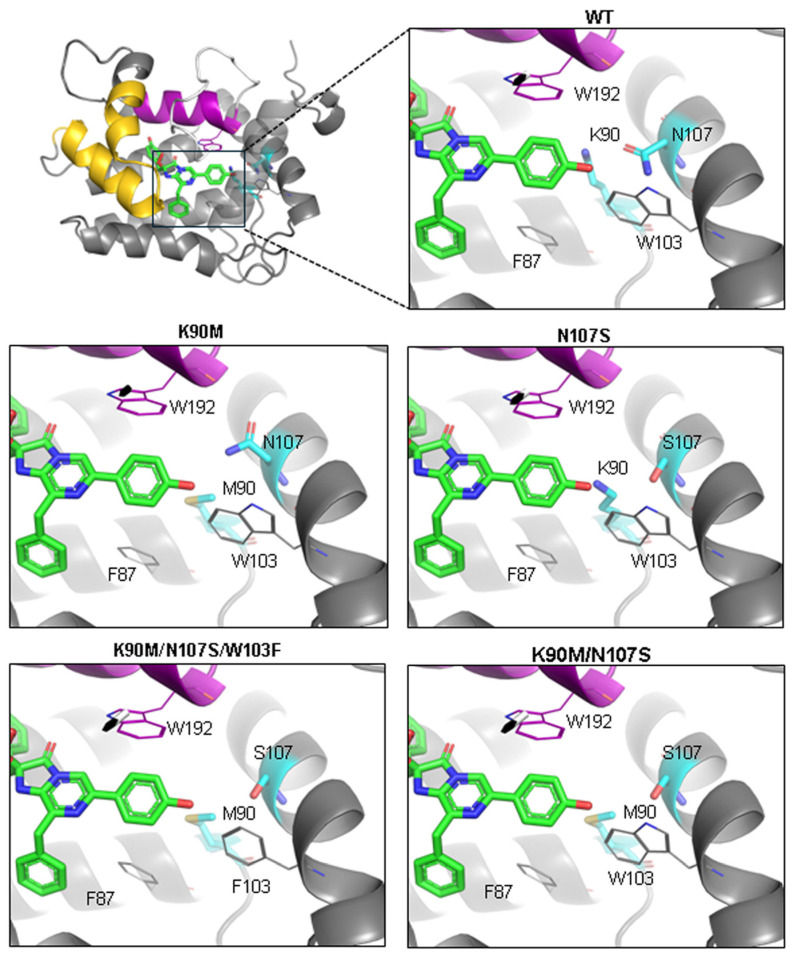
Location of side chains of the amino acid residues relative to the OH group of the 6-(*p*-hydroxy)-phenyl substituent of coelenterazine in the wild-type berovin and its mutants according to the AlphaFold model.

To explore the role of these residues in the conversion of apo-berovin into its active form, we engineered a series of mutants by altering Lys90 and Asn107. Utilizing AlphaFold’s confidence metrics, including the predicted Local Distance Difference Test (pLDDT), we selected mutations of Lys90 that minimally affected protein stability, as estimated by changes in pLDDT (Figure 2). Replacing Lys90 with Met appeared to be most suitable, as methionine has an aliphatic side chain of similar length without a polar group. To further clarify the hydrogen bond network, an additional mutation, N107S, was introduced. Since the indole group of Trp103 is situated near the OH group of the 6-(*p*-hydroxy)-phenyl substituent of coelenterazine and the Lys90/Asn107 cluster, it may hinder hydrogen bond formation by Asn107. Hence, Trp103 was also substituted to Phe.

### 3.2. Properties of Berovin Mutants

#### 3.2.1. Conversion of Apo-Berovin Mutants into Active Photoproteins

To test the effect of the predicted amino acid replacements in berovin on its properties, a set of mutants (K90A, K90M, N107S, K90A/N107S, K90M/N107S, K90A/W103F/N107S, K90M/W103F/N107S, and K90M/W103F/N107V) was constructed. The mutant apoproteins accumulated in *E. coli* cells as inclusion bodies were extracted with a buffer containing 6 M urea and purified to a homogeneous state by ion-exchange chromatography as described elsewhere [22]. Then, the apoproteins were transferred to 10 mM Tris-HCl pH 9.0 and concentrated. To determine whether substitutions affect the optimal pH and requirement of high salt concentrations for the conversion of apoprotein into active berovin, aliquots of the mutant apoproteins were added into 5 mM EDTA, 50 mM bis-Tris-propane buffer of different pH in the range from 6.0 to 9.0 without salt or with 0.5 M NaCl, containing coelenterazine (coelenterazine/apoprotein molar ratio 5:1) and incubated for 24 h at +4 °C in the dark.

**Figure 3:**
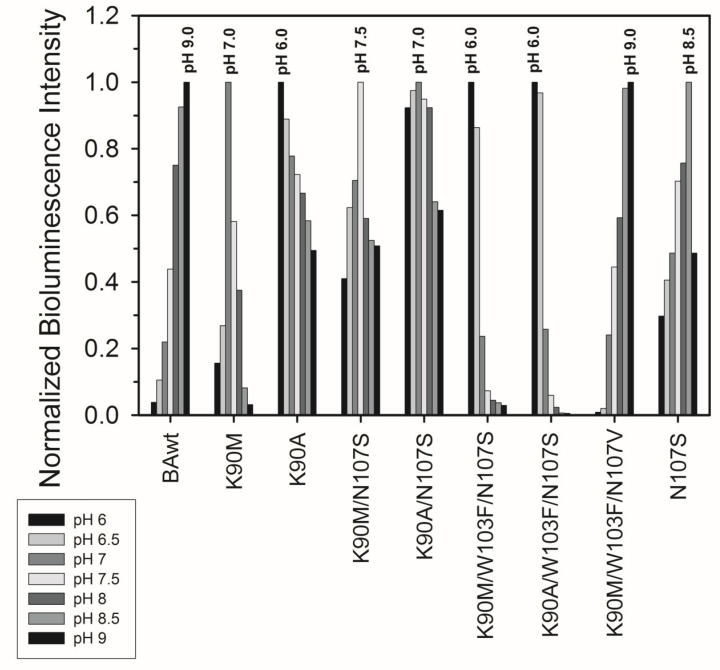
Normalized specific bioluminescence activities of berovin (BAwt) and its mutants after incubation of the corresponding apoproteins with coelenterazine (coelenterazine/apoprotein molar ratio 5:1) at different pH values with 0.5 M NaCl for 24 h at +4 °C in the dark. The specific activities at different pH values were normalized to the maximal activity for each protein.

The effect of pH on the conversion of apo-berovin and its mutants into active photoproteins is presented in Figure 3. As predicted, the replacement of Lys90 with aliphatic amino acids shifts the pH optimum towards neutral and slightly acidic pHs. The highest bioluminescence activities of K90M and K90A mutants are found after 24 h incubation at pH 7.0 and 6.0, respectively, while BAwt in this pH range shows the activity almost an order lower than that at the optimal pH of 9.0 (Figure 3). It is interesting to note that the shapes of the dependencies of bioluminescence activities on pH for K90M and K90A mutants differ. The introduction of the additional mutation N107S slightly reduces the shifts of pH optima—the highest activities of K90M/N107S and K90A/N107S mutants occurred at pH 7.5 and 7.0, respectively (Figure 3). The third additional substitution of Trp103 to Phe does not affect the shift of the pH optimum towards acidic pH; both K90A/W103F/N107S and K90M/W103F/N107S mutants revealed optima for the conversion of apoproteins into active photoproteins at pH 6.0. However, in the case of the K90A/W103F/N107V triple mutant, the pH optimum appeared to be the same as for BAwt (Figure 3), i.e., the presence of only hydrophobic residues in the environment of the 6-(*p*-hydroxy)-phenyl group of coelenterazine leads to the same effect as the presence of the side chain of Lys. The substitution of Asn107 to Ser also shifts the optimum towards acidic pH, but the shift is relatively small as compared to that for other mutants (Figure 3).

**Figure 4:**
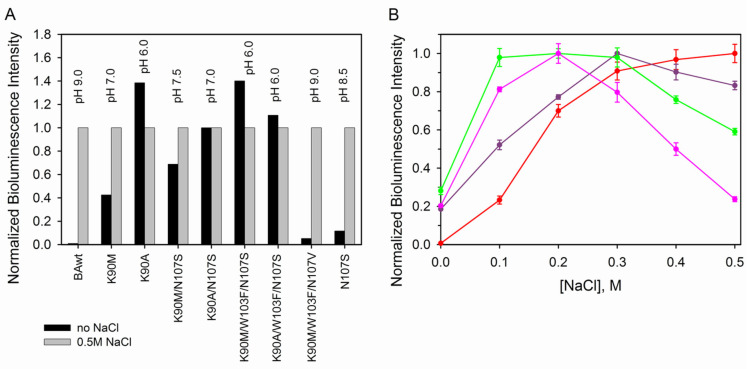
(**A**) Normalized specific bioluminescence activities of BAwt and its mutants after incubation of the corresponding apoproteins with coelenterazine at optimal pH with and without 0.5 M NaCl. The specific activities were normalized to the maximal activity for each protein with 0.5 M NaCl. (**B**) Dependence of specific bioluminescence activity of BAwt (red) and its K90M (violet), K90M/N107S (green), and K90A/N107S (pink) mutants on NaCl concentrations at optimal pH for BAwt (pH 9.0) and each mutant (pH 7.0, 7.5, and 7.0, respectively) after incubation of the corresponding apoproteins with coelenterazine. The specific activities were normalized to the maximal one for each protein. In all experiments, the bioluminescence activities were measured after incubation of corresponding apoproteins with coelenterazine (coelenterazine/apoprotein molar ratio 5:1) for 24 h at +4 °C in the dark.

Since both alkaline pH and high ionic strength are needed for an effective conversion of ctenophore apoprotein into active photoprotein we also tested the influence of introduced mutations on this process. For all mutants, activation was carried out with and without the addition of 0.5 M NaCl (which is optimal for the wild-type berovin [22]) at the optimal pH for each mutant (Figure 4A). The K90A, K90M/W103F/N107S, and K90A/W103F/N107S mutants, along with a shift of pH optima towards acidic pH, also revealed higher bioluminescent activity at low ionic strength. At the same time, in the case of the triple mutant K90M/W103F/N107V, the need for high salt concentration turned out to be critical for its conversion to the active protein like it is for BAwt. Although the substitution of Asn107 to Ser decreases the influence of salt absence on the yield of active protein as compared to the wild-type berovin, the effect is relatively small (Figure 4A). In case of K90M, K90M/N107S, and K90A/N107S mutants, the introduced substitutions reduce the effect of high salt concentration to varying degrees, suggesting the existence of optimal concentrations. Indeed, these substitutions shift the optimal ionic strength of the solution for converting apoproteins into active photoproteins toward physiological values, especially in the case of K90M/N107S and K90A/N107S mutants, for which the highest bioluminescence activities were found in the range of 0.1–0.2 M NaCl (Figure 4B).

Each mutant, at its optimal pH and NaCl concentration, was also estimated relative to the yield of active photoprotein after incubation with coelenterazine for 24 h at +4 °C in the dark. The highest yield was found for the wild-type berovin, whereas those of most mutants were ∼5–10-fold lower (Table 1). There was only one exception—the yield of the active K90M/W103F/N107S mutant appeared to be very close to that of BAwt. One of the reasons for the yield-reducing effect may be a decrease in the affinity of the substrate-binding cavity to coelenterazine. To verify this, the apparent dissociation constants (*K*_D_) were determined for the berovin mutants, which, as compared to BAwt, revealed either a lower (K90M and K90A/N107S) or comparable (K90M/W103F/N107S) yield of active protein (Table 1) [39,47]. The measurements were performed at pH and salt concentrations that are optimal for each mutant. The *K*_D_ values for K90M and K90A/N107S mutants were determined to be 2.61 ± 0.28 and 1.30 ± 0.15 μM, respectively, i.e., the affinity of the substrate-binding site of the K90M mutant to coelenterazine appeared to be equal to that for BAwt (*K*_D_ = 2.4 μM [39]) and even higher in the case of the K90A/N107S mutant. The affinity of the substrate-binding cavity of the K90M/W103F/N107S mutant (*K*_D_ = 6.62 ± 0.54 μM) turned out to be ∼2.8-fold less than that of BAwt, though the yields of active mutant and BAwt differed by less than 1.5 times (Table 1). So, there appears to be no strong correlation between the affinity of the substrate-binding site and the yield of the active photoprotein.

We also determined the *K*_D_ value for BAwt at pH 7.0 which was found to be 4.72 ± 0.36 μM, i.e., twice higher than at pH 9.0. This finding correlates well with the hypothesis proposed on the positive charge of the side chain of Lys90 hindering the formation of active photoproteins of ctenophores under physiological conditions.

#### 3.2.2. Bioluminescence Activity and Spectral Properties

Specific bioluminescence activity is an important parameter characterizing the ability of bioluminescent proteins to convert the chemical reaction energy into light. All of the mutants revealed significantly lower bioluminescent activities than the wild-type berovin (Table 1). The highest specific activity is found for the K90A mutant, and amounts to 7.7% of that of BAwt. The mutant with the replacement of the same Lys90 but with Met reveals the lowest specific activity among the mutants tested. Noteworthy is that the yields of active photoproteins for K90A and K90M mutants were practically the same (Table 1). Adding the N107S substitution to K90M increases the specific activity 67-fold, while in the K90A case the opposite effect takes place—a 17-fold drop in specific bioluminescence activity. The addition of the third substitution, W103F, slightly increases the specific activity with K90M, but reduces it by almost 2.5 times with K90A (Table 1). It is worth noting that, despite the yield of the active K90M/W103F/N107S mutant appearing to be the highest among the mutants, its specific bioluminescence activity was not only less than that of BAwt but less than that of the K90A mutant too.

Calcium ions are not strictly required for the light emission of photoproteins since they emit a very low level of light even without them. This phenomenon was named “Ca^2+^-independent luminescence” [50] and is inherent to both hydromedusan and ctenophore photoproteins. Thus, the function of calcium ions is to speed up the rate of the light emission reaction rather than to initiate it. The intensity of photoprotein Ca^2+^-independent luminescence is very sensitive to temperature [50] as well as to the substitution of amino acids [51]. Hence, the level of Ca^2+^-independent luminescence may be taken as a parameter that allows the indirect assessment of the photoprotein complex stability.

All berovin mutants showed an increased level of Ca^2+^-independent luminescence, with a varying effect of the substitutions on the intensity (Table 1). Whereas the substitution of Lys90 to Met enhances, for instance, the intensity of Ca^2+^-independent luminescence by more than 700 times as compared to that of BAwt, the replacement of this Lys with Ala does it only by ∼40-fold. The addition of the N107S mutation to K90M significantly reduces the intensity of Ca^2+^-independent luminescence of the double mutant, while in the case of the K90A mutant, introducing an additional substitution of Asn107 to Ser drastically increases Ca^2+^-independent luminescence, making the intensity comparable to that of the K90M mutant. The introduction of the third mutation W103F in K90M/N107S and K90A/N107S mutants decreases their Ca^2+^-independent luminescence, especially in the case of the K90M/W103F/N107S, in which Ca^2+^-independent luminescence appeared to be the lowest among the mutants (Table 1). The lowest level of Ca^2+^-independent luminescence may very likely be the reason for the highest yield of active photoprotein for this mutant.

The spectral characteristics of berovin mutants are summarized in Table 1. The bioluminescent spectrum of the wild-type berovin is not dependent on pH and has a maximum at 490 nm [22]. Among the constructed mutants, only N107S and K90M/W103F/N107V mutants had maxima of bioluminescence spectra at the same wavelengths as BAwt (Table 1). The bioluminescence spectra of the other mutants appeared to be shifted towards shorter wavelengths. The substitution of Lys90 to Met or Ala results in shifts of the spectral maxima by 45 and 49 nm, respectively. The introduction of an additional substitution of Asn107 to Ser in these mutants increases the blue shift of the emission spectra. The maximum of the bioluminescence spectrum of the K90M/N107S mutant was found at 415 nm with a shoulder at 500 nm. The K90A/N107S mutant revealed a spectral maximum at 402 nm with shoulders at 450 and 530 nm (Table 1). The bioluminescence spectrum of the K90M/W103F/N107S mutant had a maximum at 402 nm with a shoulder at 450 nm, i.e., its spectrum turned out to be very similar to that of the K90A/N107S mutant. The light emission with a spectral maximum at λ_max_ = 386–423 nm is attributed to the light emission from the neutral form of excited coelenteramide. The appearance of shoulders at longer wavelengths in bioluminescence spectra of some mutants clearly shows that the introduced substitutions may cause the formation of other emitting species of coelenteramide such as amide anion (λ_max_ = 435–458 nm), phenolate anion (λ_max_ = 480–500 nm), and pyrazine-N(4) anion (λ_max_ = 530–565 nm) [52]. Unfortunately, we failed to measure the bioluminescence spectrum of K90A/W103F/N107S due to the low yield of active protein and its low specific activity.

The active Ca^2+^-regulated photoproteins are non-fluorescent, but they exhibit bright fluorescence after the bioluminescence reaction. Fluorescence is conditioned by the reaction product, coelenteramide, bound within the inner cavity of the photoprotein. The fluorescence spectrum of Ca^2+^-discharged BAwt has a maximum at λ_max_ = 492 nm at pH 8.5 but is shifted towards shorter wavelengths at neutral pH (λ_max_ = 416 nm at pH 7.0). Fluorescence spectra of all Ca^2+^-discharged mutants at pH 7.0 had maxima in the range of 412–420 nm, which coincides with that of BAwt at the same pH (Table 1).

#### 3.2.3. Photo- and Thermoinactivation

**Figure 5:**
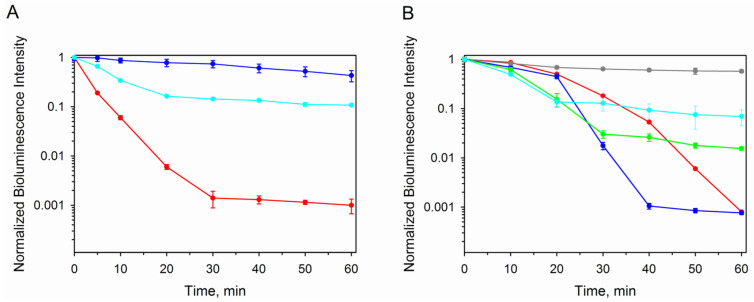
Effect of (**A**) light irradiation and (**B**) incubation at 37 °C on bioluminescence of BAwt and its mutants. (**A**) BAwt (red), K90M/W103F/N107V (cyan), and K90A (blue). (**B**) BAwt (red), K90A (blue), K90M/W103F/N107V (cyan), K90M/N107S (green), and N107S (grey).

The main distinguishing feature of ctenophore photoproteins is their ability to lose bioluminescence activity under irradiation by visible light [1]. Although the chemical structure of the photoinactivation product was recently determined [38], the mechanism and the role of residues forming the substrate-binding cavity in this process are unclear. In addition, ctenophore photoproteins are more thermolabile than those of hydromedusae [22]. Therefore, all mutants were tested for the resistance to visible light irradiation and thermal inactivation (Figure 5).

All mutants demonstrated less sensitivity to light as compared to the wild-type berovin. They can be distinguished into two groups based on their residual bioluminescent activity. The first group, including K90A, K90M, K90A/N107S, K90M/N107S, K90A/W103F/N107S, and K90M/W103F/N107S mutants, that preserves 33–46% of the initial activity after 1 h of irradiation turned out to be more numerous (Figure 5A). The second group comprises K90M/W103F/N107V and N107S mutants which retain 11% of the initial activity, thereby indicating their lower resistance to light irradiation. Noteworthy is that the optimal conditions for the conversion of these mutants into active photoproteins were found at alkaline pH and high ionic strength, i.e., similar to those for the wild-type apo-berovin (Figure 3 and Figure 4).

The effects of mutations on berovin thermoinactivation are more diverse when compared to photoinactivation (Figure 5A). All mutants, based on the residual activity after 1 h incubation at 37 °C, may be distinguished into four groups. The mutant with the replacement of Lys90 with Ala loses activity even faster than the wild-type berovin. At the same time, the residual activity of both BAwt and K90A mutants appeared to be equal (0.08%) to the initial activity. The N107S mutant revealed the highest resistance to thermal inactivation, retaining 60% of the initial activity after 1 h incubation at 37 °C. The K90A/W103F/N107S and K90M/W103F/N107V mutants were also more stable as compared to BAwt, preserving 6 and 7% of activity, respectively. The most numerous group consists of the K90M, K90A/N107S, K90M/N107S, and K90M/W103F/N107S mutants, with 0.5–1.6% of initial activity retained (Figure 5B).

## 4. Conclusions

In summary, we have performed the first study on the identification of the residue located within the substrate-binding cavity of ctenophore photoproteins that accounts for the formation of an active photoprotein from apoprotein and coelenterazine at alkaline pH and high ionic strength. Based on the model of berovin spatial structure with the 2-hydroperoxy adduct of coelenterazine obtained by AlphaFold, we highlighted a cluster of polar residues consisting of Lys90 and Asn107 within a hydrophobic environment. According to the model, these residues might also form hydrogen bonds with the OH group of the 6-(*p*-hydroxy)-phenyl substituent of coelenterazine. Considering that Lys90 is positively charged at neutral pH and is neutral at alkaline pH, this cluster was proposed to be responsible for the requirement of alkaline pH to convert apo-berovin into active photoprotein. Indeed, the substitution of Lys90 to Ala (K90A) or Met (K90M) resulted in a shift of the pH optimum for the conversion of apo-berovin into active photoprotein towards physiological pH (Figure 3). However, these replacements significantly decreased the yield of active protein and specific activity, and increased the Ca^2+^-independent luminescence, thereby indicating the reduction in photoprotein stability (Table 1). It should be pointed out that Met is found in the substrate-binding cavity of both obelin and aequorin in the same place and orientation related to the 6-(*p*-hydroxy)-phenyl substituent of coelenterazine as Lys in berovin, and the Met side chain interacts with the phenyl ring through π-alkyl stacking [53]. Lys might function like Met does in hydromedusan photoproteins but only at alkaline pH values. To compensate for the distortions caused by substitutions, we introduced additional mutations utilizing AlphaFold’s confidence metrics, including the predicted Local Distance Difference Test. The triple mutant K90M/W103F/N107S revealed the increased yield of active photoprotein under physiological pH and ionic strength and decreased level of Ca^2+^-independent luminescence, but specific bioluminescent activity turned out to still be low, i.e., the sufficient yield of the active protein does not yet guarantee its high bioluminescent-specific activity. It is also interesting to note that all of the mutants revealed higher resistance to light irradiation as compared to the wild-type berovin. Although we failed to produce berovin mutants with high specific bioluminescent activity that would be converted into active photoprotein under physiological conditions, our findings are nevertheless quite important since we succeeded in localizing the key residue responsible for this property. Since the amino acids that form the substrate-binding cavity of the ctenophore photoproteins are completely different from those forming the inner cavity of the hydromedusae photoproteins, one would hardly expect that a substitution of only three residues would convert the ctenophore photoprotein into something similar to hydromedusan photoproteins.

The animals forming the Ctenophora phylum represent the descendants of the earliest surviving lineage of ancestral metazoans. Numerous modern studies demonstrate that many aspects of ctenophore biology, as well as their development, are significantly different from those described in the representatives of the other 32 animal phyla, thereby implying that most system-level components (neurons, synapses, muscles, mesoderm, etc., for example) related to ctenophore organization are the result of convergent evolution [54]. Most likely, Ca^2+^-regulated photoproteins of the ctenophore are also its products, as in the other ctenophore systems. Moreover, it cannot be ruled out that during evolution, specialized cells (photocytes, where photoproteins are localized) supporting alkaline pH either during a short time as a response to light irradiation or mechanical stimuli, for example, or permanently, would also arise, thereby providing the bright bioluminescence of ctenophores.

## Figures and Tables

**Table 1:** Properties of berovin mutants.

Photoprotein	Yield of Active Protein, %	*L*_specific_, RLU, ×10^9^	*L*_Ca-free_, RLU, ×10^−8^	BL λ_max_/ Shoulder λ_max_, nm	FL λ_max_/ Shoulder λ_max_, nm
Berovin (BAwt)	65 ± 5	300 ± 0.22 (100%)	0.4	**490** *	**492**/398 (pH 8.5)
					**416** (pH 7.0)
K90A	10 ± 1	23.0 ± 0.23 (7.7%)	17	**441**	**418**
K90M	14 ± 1	0.09 ± 0.003 (0.03%)	300	**445**	**412**
N107S	13 ± 1	1.4 ± 0.06 (0.47%)	71	**490**	**428**
K90A/N107S	8 ± 1	1.3 ± 0.2 (0.44%)	480	**402**/450/530	**420**
K90M/N107S	10 ± 1	6.0 ± 0.13 (2.0%)	58	**415**/500	**420**
K90A/W103F/N107S	8 ± 1	0.54 ± 0.027 (0.18%)	37	ND **	**416**
K90M/W103F/N107S	47 ± 2	7.0 ± 0.98 (2.3%)	8.1	**402**/450	**415**
K90A/W103F/N107V	7 ± 1	1.1 ± 0.027 (0.37%)	77	**490**	**425**

* Main peak is shown in bold; ** ND, not detected.

## Data Availability

The original contributions presented in the study are included in the article, further inquiries can be directed to the corresponding authors.
